# High Hydrostatic Pressure Processing of Whole Carrots: Effect of Static and Multi-Pulsed Mild Intensity Hydrostatic Pressure Treatments on Bioactive Compounds

**DOI:** 10.3390/foods10020219

**Published:** 2021-01-21

**Authors:** Fernando Viacava, Perla A. Ramos-Parra, Jorge Welti-Chanes, Daniel A. Jacobo-Velázquez

**Affiliations:** 1 Tecnologico de Monterrey, Escuela de Ingeniería y Ciencias, Ave. Eugenio Garza Sada 2501, Monterrey, Nuevo Leon 64849, Mexico; viacavaromo@gmail.com (F.V.); perlaramos@tec.mx (P.A.R.-P.); jwelti@tec.mx (J.W.-C.); 2 Tecnologico de Monterrey, Escuela de Ingeniería y Ciencias, Ave. General Ramón Corona 2514, Zapopan Jalisco 45201, Mexico

**Keywords:** high hydrostatic pressure (HHP) processing, multi-pulsed HHP, elicitation, phenolic compounds, carotenoids

## Abstract

In this study, the effects of static and multi-pulsed mild-intensity high hydrostatic pressure (HHP) treatments (60 or 100 MPa, ~23 °C) on the extractability and accumulation of phenolics and carotenoids in whole carrots were evaluated. HHP treatments were applied for the time needed to reach the desired pressure (come-up-time, CUT) either as a single pulse or multi-pulse (2P, 3P, and 4P). Likewise, a single sustained treatment (5 min) applied at 60 or 100 MPa was evaluated. Individual carotenoids, free and bound phenolics were quantified after HHP treatment and subsequent storage (48 h, 15 °C). As an immediate HHP response, phenolic extractability increased by 66.65% and 80.77% in carrots treated with 3P 100 MPa and 4P 60 MPa, respectively. After storage, CUT 60 MPa treatment accumulated free (163.05%) and bound (36.95%) phenolics. Regarding carotenoids, total xanthophylls increased by 27.16% after CUT 60 MPa treatment, whereas no changes were observed after storage. Results indicate that HHP processing of whole carrots at mild conditions is a feasible innovative tool to enhance the nutraceutical properties of whole carrots by increasing their free and bound phenolic content while maintaining carotenoid levels. HHP treated carrots can be used as a new functional food or as raw material for the production of food and beverages with enhanced levels of nutraceuticals.

## 1. Introduction

Horticultural crops contain high levels of health-promoting compounds, also known as nutraceuticals [1]. These nutraceuticals are plant secondary metabolites, which accumulation can be induced by the application of controlled postharvest abiotic stresses to break homeostasis [2]. Wounding is the most effective abiotic stress to activate the secondary metabolism of horticultural crops, leading to the accumulation of nutraceuticals [3]. However, the application of wounding stress as a tool to generate fresh produce with enhanced levels of antioxidants has some limitations mainly due to its detrimental effects on quality during shelf-life [4]. Moreover, after the unit operation of cutting is applied to obtain the fresh-cut product, in most cases a post-wounding sanitizing procedure is required, which includes dipping the tissue in sanitizing solutions, resulting in a decrease or elimination of the primary wound-signal (extracellular ATP) that induces the wound-response in plants [5].

In this context, based on previous reports mainly using plant cell cultures, we recently proposed that nonthermal processing technologies such as ultrasound, high hydrostatic pressure (HHP) processing and mild-intensity pulsed electric fields could emulate a wound-like stress response in horticultural crops [6,7,8]. This wounding-like response induces the secondary metabolism of horticultural crops leading to the accumulation of nutraceuticals, while decreasing the drawbacks of wounding stress application on the quality of fresh produce. This concept was validated using carrots as a model system, where ultrasound [9], HHP processing [10] and mild-intensity pulsed electric fields [11] were applied to increase the concentration of antioxidant phenolic compounds. Likewise, the concept was also proven in broccoli, where the application of ultrasound in combination with phytohormones increased the concentration of phenolics, glucosinolates, vitamin C and isothiocyanates in the crop [12].

Recently, it was reported that HHP processing come-up time (CUT, 60–100 MPa), which requires less processing time and energy cost, could be used as an innovative tool to increase the extractability and induce the biosynthesis of free and bound phenolic compounds in carrots during storage (3 d at 15 °C) [10]. Benefits of using HHP as an abiotic elicitor of secondary metabolites include its potential to inactive detrimental enzymes and microorganisms in the tissue and induce the biosynthesis of nutraceuticals in whole tissues emulating the wound-like response; whereas a drawback could be that in an attempt to inactivate undesirable enzymes, key enzymes involved on the biosynthesis of the bioactive compound could also be inactivated [8]. Thus, it is relevant to evaluate the effect of additional HHP processing conditions, such as static and multi-pulsed mild intensity pressure treatments, on the biosynthesis and accumulation of free and bound phenolics, as well as on the carotenoids, which are one of the major bioactive compounds present in carrots. HHP treated carrots could be used as a new functional food or as raw material for the production of food and beverages with enhanced levels of nutraceuticals.

Therefore, the objective of this study was to evaluate the effects of different static and multi-pulsed mild intensity pressure treatments (60 or 100 MPa, ~23 °C) on the extractability and biosynthesis of phenolic compounds and carotenoids in whole carrots immediately after processing and after storage (48 h at 15 °C).

## 2. Materials and Methods

### 2.1. Reagents

Ethanol (HPLC grade), methanol (HPLC grade), acetone (HPLC grade), methyl tert-butyl ether (MTBE, HPLC grade) and hydrochloric acid [(HCl) 98% *v/v*] were obtained from Fisher Scientific (Waltham, MA, USA). Ethyl acetate, hexane, sodium hydroxide (NaOH), and orthophosphoric acid [(H3PO4) 0.097% *v/v*] were acquired from Desarrollo de Especialidades Químicas (San Nicolás de los Garza, NL, México). Mili-Q^®^ ultrapure water was obtained from a purification system (Ultrapure and Pure Water System^®^, Merck, Darmstadt, Germany). Chlorine (Cloralex^®^, 6% sodium hypochlorite) was purchased from a local supermarket (HEB, Monterrey, México). High barrier pouches (27 × 35 cm) made of nylon and low-density polyethylene (water permeability, <1.2g × 645 cm^−2^ 24 h^−1^, at 37 °C and 100% RH), used for vacuum packaging carrots before HHP treatment, were acquired from Filmpack^®^ (Guadalupe, NL., México). All other chemicals were obtained from Sigma-Aldrich^®^ (St. Louis, MO, USA).

### 2.2. Plant Material and High Hydrostatic Pressure (HHP) Conditions

Carrots were obtained from a local market (HEB, Monterrey, México). Prior to HHP treatments, carrots were washed with tap water, sanitized with a chlorine solution (200 ppm, pH 6.5–7.0) for 5 min, and dried using a paper towel. Sanitized carrots were vacuum-packed (67.7 kPa) in pairs using high barrier pouches and a Multivac R230 series 542 (Multivac, Wolfertschwenden, Germany). High hydrostatic pressure was applied using a pressurization system (Avure Technologies Inc., Middletown, OH, USA), equipped with a 2 L chamber. Tap water was used as pressure transmission fluid. Temperature inside the chamber varied from 21.5 °C to 24.27 °C depending on the pressure treatment applied.

The immediate and late response of different HHP conditions, on the content of bioactive compounds, was evaluated. HHP conditions evaluated were: come-up-time (CUT) as the time needed to reach the expected hydrostatic pressure, two pulses (2P), three pulses (3P), four pulses (4P) and sustained pressure for 5 min, each at 2 levels of hydrostatic pressures: 60 and 100 MPa. Pressurization rate was 4.06 MPa s^−1^ over 15.33 ± 2 s to reach 60 MPa and 4.61 MPa s^−1^ for 20.67 ± 2 s to reach 100 MPa. Decompression of the chamber was instantaneous (<1 s). For storage studies, samples were removed from vacuum-sealed bags after processing and placed in hermetic plastic containers (3.8 L). To keep the temperature constant (15 °C) containers were maintained in a Symphony incubator (VWR, Radnor, PA, USA) for 48 h. For each HHP treatment and the control group, 6 carrots of ~130 g each were used per replicates. Three independent experiments were conducted. Carrots were collected at 0 h and 48 h to quantify individual free and bound phenolics as well as individual carotenoids.

### 2.3. Phytochemical Analysis

#### 2.3.1. Identification and Quantification of Free and Bound Phenolics

Free and bound phenolic compounds in samples were extracted and identified using the method described by Viacava et al. [10,13]. Briefly, 4 g fresh weight (FW) was blended thoroughly with an ethanol solution (80%, 10 mL) and a tissuemizer (Advanced Homogenizing System, VWR). Free phenolics were separated from the mixture by centrifugation (12,000× *g*, 20 min), which remained in the supernatant while the bound phenolics were extracted from the pellet (carrot solids). To remove the solvents, samples were carefully dried using a vacuum evaporator (EZ-2.3, Genevac Ltd. Ipswich, EN). Samples were dissolved using a methanol solution (50% *v/v*, 2 mL) prior to filtration using a nylon membrane (0.45 μm, VWR) and chromatographic analysis.

Bound phenolics were extracted from free phenolic precipitate using 10 mL of 2 M NaOH and high temperature (95 °C) as indicated by Viacava et al. [13]. The hydrolysis reaction of bound phenolics linkages was stopped by neutralization with concentrated HCl. Lipids were removed with hexane and discarded. Bound phenolics were recovered with ethyl acetate and concentrated to dryness at low temperature (35 °C) under vacuum conditions (EZ-2.3, Genevac Ltd. Ipswich, England). Finally, samples were resuspended in methanol (50% *v/v*, 2 mL), filtered (0.45 μm, VWR) and analyzed with a HPLC-DAD 1260 Infinity System (Agilent Technologies, Santa Clara, CA, USA). The determination of individual phenolics was performed as previously described by Viacava et al. [10,13].

Free and bound phenolics profiles from carrot samples were assessed by chromatographic separation using a C18 reverse-phase column with dimensions 4.6 mm x 250 mm, 5 µm particle size (Luna, Phenomenex, Torrance, CA, USA) with its corresponding C18 guard cartridge. Mobile phases were composed of water (phase A) and methanol:water (60:40, v:v, phase B) both adjusted to pH 2.4 with ortophosphoric acid. Injection volumes were 10 µL for each replicate and the solvent gradient to elute the compounds was 0/100, 3/70, 8/50, 35/30, 40/20, 45/0, 50/0, 60/100 (min/% phase A) with a flow rate of 0.8 mL min^−1^. The identification of individual phenolic acids was performed by comparing their absorption spectrum with authentic standards. The detector was a diode array programmed to record signals at 280, 320 and 360 nm. For quantification of individual free and bound phenolic compounds, standard curves were performed (0.25–500 ppm). Finally, phenolic concentration was calculated as mg kg^−1^ dry weight (DW). The moisture content (%) of the samples was determined by the air–oven method (AACC 44-15A) at time 0 day, as well as the weight loss of samples, which was obtained during storage, in order to calculate the percentage (%) of moisture content.

#### 2.3.2. Identification and Quantification of Carotenoids

Carotenoids were evaluated as indicated by Viacava et al. [13]. Extractions were made under dark conditions and room temperature (21 °C). Samples of 2 g fresh weight (FW) were homogenized with 20 mL of acetone (added with 200 mg L^−1^ BHT) for 30 s using a tissuemizer (Advanced homogenizing system, VWR). Homogenates were filtrated through a Whatman No. 1 filter paper under vacuum. This procedure was repeated 5 times for each sample to ensure complete color extraction. Filtered extracts were collected on a 100-mL volumetric flask and volume was completed with the acetone solution. Extracts were filtered with PTFE membranes (0.45 μm, Millipore, Billerica, MA, USA) and placed in vials whose headspaces were filled with nitrogen to prevent oxidation of carotenoids.

Carotenoids were separated on a C30 reverse-phase column (4.6 × 150 mm, 3 μm particle size) (YMC, Wilmington, NC, USA), coupled to a corresponding C30 guard cartridge. Vial chamber and column temperatures were 4 and 30 °C, respectively. The mobile phase consisted of 50% methanol, 45% MTBE, and 5% water. Injection sample volume was 25 μL. The system was isocratic, where total elution time was 25 min at a flow rate of 0.5 mL min^−1^. Carotenoids were recorded at 450 nm and individual quantification was carried out with standard curves from β-carotene (0.1–30 ppm) for carotenoids and lutein (0.125–12 ppm) for xanthophylls. The identification of carotenoids was performed by comparison of elution order and maximum absorption wavelength (λmax) with those reported in previous reports [13,14].

### 2.4. Statistical Analysis

Results were expressed as mean values ± standard error of the mean. To evaluate significant statistical differences between means a one-way ANOVA was carried out, followed by a Tukey test (*p* < 0.05). Likewise, to compare between treatments at different storage times Student’s *t*-test pair comparisons were performed using Minitab software (Minitab 19, Minitab Inc., State College, PA, USA).

## 3. Results

### 3.1. Phenolics

**Figure 1:**
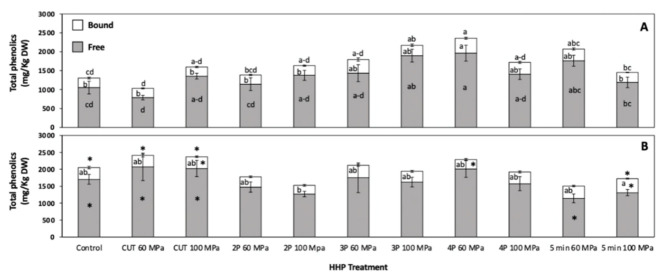
Concentration of free, bound, and total phenolics before (**A**) and after storage (48 h at 15 °C, **B**) of whole carrots treated with static and multi-pulsed mild intensity pressure treatments. Different letters within bars indicate significant differences between free and bound phenolic content among samples. Different letters at the top of the bars indicate significant difference in the total phenolic content between treatments. Asterisk (*) indicates statistical difference comparing values of each stored sample with its corresponding value before storage by Student’s *t*-test (*p* < 0.05). Values represent the mean of three replicates and their respective standard error. Statistical significance between means was calculated by one-way analysis of variance, followed by a Tukey’s test (*p* < 0.05).

The total phenolic content in whole carrots divided as free and bound phenolics is shown in Figure 1. As an immediate response to HHP, carrots treated with 3P 100 MPa and 4P 60 MPa showed an immediate increase in total phenolics (Figure 1A). These increases were mainly associated with the increased quantification of free phenolic compounds after HHP treatment, where samples treated with 3P 100 MPa and 4P 60 MPa showed increases by 79.93% and 86.14%, respectively, as compared with the control. Likewise, for the 4P 60 MPa treatment, the total bound phenolics showed an increase of 58.00%. All other treatments did not show a significant difference in totally free and bound phenolics as compared with the control.

Stored samples did not show a significant difference in the total phenolic content between the control and high hydrostatic pressure (HHP) treated samples (Figure 1B). However, when comparing stored samples (Figure 1B) against samples before storage (Figure 1A), the control and samples treated under low-intensity HHP conditions (CUT 60 MPa and CUT 100 MPa) showed significant increases in the concentration of phenolic compounds. For instance, CUT 60 MPa showed a significant raise of total free (163.05%) and bound (36.95%) phenolics and their sum (133.13%) as compared with their corresponding control before storage.

#### 3.1.1. Free Phenolics

The free phenolics compounds identified included gallic acid hexoside (GAH), 3-*O*-caffeoylquinic acid (3-*O*-CQA), 5-*O*-caffeoyquinic acid (5-*O*-CQA), 3,5-di-*O*-caffeoylquinic acid (3,5-diCQA), 4,5-di-*O*-caffeoylquinic acid (4,5-diCQA), ferulic acid, 3,4-*O*-diferuloyquinic acid (3,4-diFQA) and isocoumarin. The free phenolic compounds identified agree with previous reports on the identification of free phenolic profiles in carrots [10,13].

When comparing the control against HHP processed samples, 3,4-diFQA was the free phenolic that showed a marked increase after pressurization (Table 1). This increase was more intense as the pressure treatment increased. For instance, 3P 100 MPa was the treatment that showed the highest immediate increase (1747.9%) in 3,4-diFQA concentration after pressurization.

After storage, samples treated with HHP showed a significant increase in GAH, ferulic acid, 3,4-diFQA and isocoumarin as compared with the control before storage (CBS). The increases in GAH were detected in samples treated with CUT 60 MPa (97.45%) and 4P 60 MPa (149.69%). On the other hand, carrots treated with CUT at 60 MPa and 100 MPa showed increases in ferulic acid by 120.16% and 175.14%, respectively, as compared with CBS. Regarding 3,4-diFQA, all HHP treated samples showed a significant increase in its concentration, where samples treated with CUT 100 MPa presented the highest increase (2080.45%). Finally, the samples treated with 2P 60 MPa showed 60.42% of increase in isocoumarin content as compared with CBS (Table 1).

#### 3.1.2. Bound Phenolics

The effect of HHP and storage time on the content of individual bound phenolic compounds in whole carrots is shown in Table 2. The bound phenolics identified included caffeoyl glucose, 4-*O*-coumaroylquinic acid (4-*O*-CoQA), 5-*O*-coumaroylquinic acid (5-*O*-CoQA), caffeic acid, and *p*-coumaric acid, rutin and quercetin. The bond phenolic profile identified in whole carrots is in agreement with previous reports [10,13].

As an immediate response to HHP, the content of *p*-coumaric acid increased by 124.7% in samples treated with 4P 60 MPa, whereas the levels of the other bound phenolics remained unaltered. After storage (48 h at 15 °C) the bound phenolics that showed an increase in HHP treated samples as compared with CBS were the caffeoyl glucose, 4-*O*-CoQA, and *p*-coumaric acid. For caffeoyl glucose, the treatment that showed a significant increase (84.98%) as compared with CBS was the whole carrots treated with 100 MPa for 5 min. On the other hand, the 4-*O*-CoQA showed increases by 150.74%, 127.6%, and 68.65%, for whole carrots treated with 3P 60 MPa, 5min 60 MPa, and CUT 100 MPa, respectively, as compared with CBS. Finally, the *p*-coumaric acid increased by 68.65%, and 99.19% for 3P 60 MPa and CUT 60 MPa, respectively, as compared with CBS; whereas whole carrots treated with 100 MPa increased by 79.06%, 45.97%, 76.9%, and 43.88% as compared with CBS, for the sample treated under that pressure for the CUT, 2P, 3P, and 4P, respectively (Table 2).

### 3.2. Carotenoids

**Figure 2:**
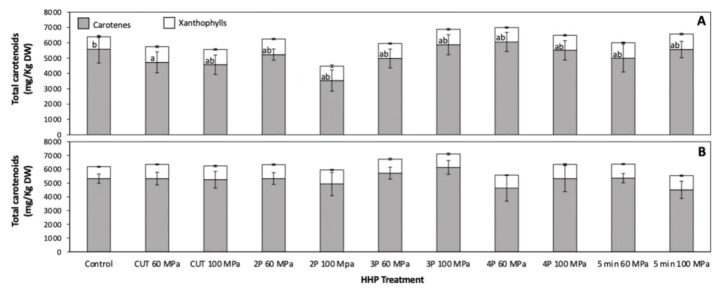
Concentration of carotenes, xanthophylls, and total carotenoids before (**A**) and after (**B**) storage (48 h at 15 °C) of whole carrots treated with high hydrostatic pressure processing (HHP). Different letters inside within bars indicate significant differences between xanthophyll content among samples before storage. Values represent the mean of three replicates and their respective standard error. Statistical significance between means was calculated by one-way analysis of variance, followed by a Tukey’s test (*p* < 0.05).

The total carotenoid content of whole carrots treated and non-treated with HHP is shown in Figure 2. As observed, the total carotenoid content was not affected by HHP treatments. However, the total xanthophylls showed a slight increase (27.16%) in samples treated with CUT 60 MPa. After storage (48 h at 15 °C), a non-significant difference was detected in the content of carotenes, xanthophylls and total carotenoids between HHP treated stored samples (Figure 2B). Likewise, no significant difference was observed in the carotenoid content when comparing stored samples (Figure 2B) against samples before storage (Figure 2A).

#### Individual Carotenes and Xanthophylls

Individual carotenoids detected in whole carrots included lutein, zeaxanthin, β-cryptoxanthin, α-carotene and β-carotene, all of them in their all-*trans* molecular configuration (Table 3). The carotenes (α-carotene and β-carotene) contributed with 87.45% of total carotenoids, whereas the xanthophylls with 12.55%. The carotenoid profile reported herein agrees with previous reports [13,15].

The content of individual carotenes and xanthophylls as affected by static and multi-pulsed mild intensity HHP treatments in whole carrots is shown in Table 3. As an immediate response to HHP treatment, the content of lutein increased by 18.63% in samples treated with 3P 60 MPa, whereas all other pressure treatments did not affect lutein content. Likewise, zeaxanthin content increased as an immediate response to 60 MPa CUT (86.68%), 2P (76.25%), and 3P (71.60%) and 1P/5 min (73.18%) treatments; whereas 100 MPa CUT, 2P, 3P, 4P and 1P/5 min also increased zeaxanthin content by 75.72%, 74.86%, 71.25%, 72.79%, 77.83%, respectively, where no significant difference was detected between 60 and 100 MPa treatments. For β-cryptoxanthin, its content immediately increased after 60 MPa CUT (75.79%), 2P (68.15%), and 1P/5 min (65.07%); and 100 MPa CUT (66.67%), 3P (70.08%), and 1P/5 min (63.71%) treatments. Regarding carotenes (α- and β- carotene), HHP treatments only affected the content of α-carotene (−38.69%) in whole carrots as an immediate response to 2P 100 MPa treatment, and thus the RAE was not significantly changed.

After storage, the content of lutein remained higher (11.75%) in 3P 60 MPa treatment as compared with the stored control, whereas no significant difference was detected between stored HHP treated carrots and samples before storage. Regarding zeaxanthin content, the CUT 60 MPa and 2P 100 MPa showed 42.19% and 42.9%, respectively, higher levels as compared with the stored control samples; whereas all HHP treated samples showed higher zeaxanthin content than the CBS. However, a non-significant difference was detected in the stored HHP treated samples as compared with samples before storage. A similar trend was observed for β-cryptoxanthin content quantified after storage in HHP treated samples, where the 60 MPa CUT and 1P/5min treatments showed 31.21% and 33.41%, respectively, higher β-cryptoxanthin content as compared with the stored control. Likewise, the 100 MPa 2P, 4P and 1P/5min showed 32.75%, 42.82%, and 38.04%, respectively, higher β-cryptoxanthin content as compared with the stored control. Regarding carotenes (α- and β- carotene) content, no significant differences were detected between HHP treated samples and the control after storage.

## 4. Discussion

HHP is commonly applied in foods to induce microbial and enzymatic inactivation and enhance the shelf-life of processed products, while preventing undesirable changes in the sensory, nutritional and physicochemical properties [7]. However, the is scarce information on the effect of HHP as an abiotic elicitor to induce the biosynthesis of health-promoting compounds. It is proposed that mild intensity HHP treatments (<150 MPa) induce a stress response in plants, while high-intensity HHP treatments (>150 MPa) induce irreversible damage in plant cells or even plant cell death [16]. In this context, in the present study, the effect of mild intensity HHP treatments (60 MPa and 100 MPa) applied as multi-pulse (CUT, 2P, 3P and 4P) and for as a single static pulse maintained for 5 min, on the content of free and bound phenolics, and on carotenoid content in whole carrots was evaluated.

### 4.1. Immediate Response of Whole Carrots to Static and Multi-Pulsed Mild Intensity Pressure Treatments

The stress-responses in plant tissues can be divided as immediate and late stress responses [5,6,16]. In the specific case of HHP, immediate plant cell stress responses include: (1) increased extractability of bioactive compounds and increased biosynthesis due to HHP-induced enzyme activation, and (2) increased production of primary and secondary stress signaling molecules that activate the biosynthesis of secondary metabolites as a late stress response [6,7,10,16,17,18,19,20,21]. In the specific case of phenolics, HHP generated increased quantification of free and bound phenolics in whole carrots, where a higher number of HHP pulses at 60 and 100 MPa generated higher levels of phenolics. On the other hand, HHP increased the content of xanthophylls, where the major concentrations were detected at 60 MPa CUT. Likewise, carotene contents were not affected by HHP treatments.

As earlier described, the higher quantification of bioactive compounds is one of the early stress responses of horticultural crops to mild intensity HHP treatments. Thus, higher levels of free and bound phenolics, and xanthophylls can be attributed to higher extractability and stress-induced biosynthesis of secondary metabolites [6,19]. Higher extractability could be the result of cell membrane disruption as previously reported for crops such as prickly pear [20,21], baby carrot [22], and onion [23]. Likewise, HHP could be disrupting chemical interactions between macromolecules and secondary metabolites, enhancing its extractability. Phenolics and carotenoids can be found in free and bound forms in plant tissues. Phenolics are mainly linked to cell-wall components such as polysaccharides; whereas carotenoids (mainly xanthophylls) are bound to proteins or esterified to fatty acids [24,25]. Chemical interactions attaching secondary metabolites to cell-wall components and other macromolecules include noncovalent bonds (van der Waals attractions, hydrogen bonds, dipolar interactions, and electrostatic) between polar groups from proteins or polysaccharide molecules and hydroxyl groups from phenolics or carotenoids [24,25,26,27]. HHP disrupt all these types of chemical interactions, increasing the extractability of secondary metabolites [28]. As observed in the results from the present study, xanthophylls (lutein, zeaxanthin, and β-cryptoxanthin) showed higher quantification after HHP treatment, whereas the carotene content remained unaltered. This can be explained by the difference in the chemical structure of carotenes and xanthophylls. Whereas xanthophylls have hydroxyl groups that allow their chemical interaction with macromolecules, carotenes are mainly in the free form [14,17,25]. Likewise, the main phenolic that increased was the 3,4-diFQA, which is a hydroxycinnamic acid attached to cell-wall components (i.e., lignin and cellulose) through hydrophobic forces and hydrogen bonding [3]. Therefore, these results indicate that xanthophylls and 3,4-diFQA were released from macromolecules after pressurization, increasing their extractability.

An additional explanation for increased quantification of hydroxycinnamic acids and xanthophylls as an immediate response to HHP, could be the pressure-induced biosynthesis of secondary metabolites. This phenomenon has been previously reported for papaya, where the application of HHP treatment increased the gene expression and accumulation of carotenoids as an immediate response to pressurization [17]. Similar results to the one reported herein for free and bound phenolics were previously observed by Viacava et al. [10], where the application of HHP at 60 MPa for the CUT immediately increased the enzymatic activity of phenylalanine ammonia-lyase (PAL), which is the key enzyme in phenolics biosynthesis, confirming the immediate pressure-induced activation of phenolics biosynthesis in whole carrots.

### 4.2. Late Response of Whole Carrots to Static and Multi-Pulsed Mild Intensity Pressure Treatments

As a result of the production of primary and secondary stress signaling molecules in the immediate response to HHP, the secondary metabolism of plants is activated leading to the accumulation of secondary metabolites as a late stress response. In the present study, the accumulation of free and bound phenolics was detected after 48 h of storage at 15 °C. On the other hand, HHP treated whole carrots did not show significant accumulation of carotenoids, although stored HHP treated samples showed higher levels of carotenoids as compared with the stored control.

The accumulation of free and bound phenolics in HHP treated whole carrots has been associated with a wounding-like response [6,7,8,10]. Upon wounding-stress application, extracellular adenosine triphosphate (eATP) is released from the cytoplasm of wounded cells, bounding to ATP receptors of adjacent cells [5]. ATP is the primary signal that increases cellular respiration, reactive oxygen species (ROS) production, and ethylene [9]. ROS and ethylene serve as a secondary wound signal that activates the biosynthesis of phenolics in carrots. In this context, according to previous reports [6,10,17], it is hypothesized that HHP induces cell membrane disruption, promoting the release of ATP from the damaged cell and the subsequent production of secondary signals as described for wounding. The main free and bound phenolics accumulated are hydroxycinnamic acids, which are the main phenolics accumulated due to wounding. These compounds serve as lignin precursors, which are produced to prevent water-loss during the wound-healing process [29]. This observation supports the hypothesis that HHP induces a wounding-like response in horticultural crops. It is likely that upon the application of wounding stress, enzymes such as polyphenol oxidase (PPO) and peroxidase (POD) star to oxidize phenolic compounds, which are their substrate [29]. Interestingly, herein, we did not observe the degradation of these compounds and rather phenolic compounds were accumulated. This can be attributed to a higher biosynthesis rate as compared with their oxidation rate catalyzed by both enzymes (PPO and POD) [29].

## 5. Conclusions

In this study, a multi-pulse HHP approach was evaluated as a potential strategy to induce the biosynthesis and accumulation of bioactive compounds in whole carrots. To the best of our knowledge, this is the first report in the literature that evaluated the effects of multi-pulse HHP treatments at physiological pressures (60 and 100 MPa) on the content of carotenoids and phenolics in whole carrots. The results showed higher total phenolics (free and bound) as the intensity of pressure and number of pulses incremented. As expected, whole carrots subjected to more stages of pressurization showed higher extractability of phytochemicals. On the other hand, lower pressure intensities elicited the biosynthesis of free and bound phenolics. The results presented herein, indicate that HHP could be used as an innovative tool to obtain the next generation of fresh produce that could be consumed as fresh food, as raw material for the production of processed foods or as a primary source of valuable natural antioxidants. Further studies should evaluate the shelf-life stability of HHP-treated whole carrots under the selected processing conditions, to validate the feasibility of incorporating the product in the fresh-produce market. Variables such as instrumental color change, microbiological analyses, sensory acceptability, texture analyses and enzymatic activities (i.e., PPO and POD) should be included in the aforementioned shelf-life study.

## Figures and Tables

**Table 1:** Effect of static and multi-pulsed mild intensity pressure treatments on the concentration of free phenolics in whole carrot samples.

Treatment			Phenolic Concentration (mg/kg DW) ^1,2,3,4,5,6,7^							
Storage Time	Pressure	Number of Pulses	GAH	3-*O*-CQA	5-*O*-CQA	3,5-diCQA	4,5-diCQA	Ferulic Acid	3,4-diFQA	Isocoumarin
	Applied (MPa)									
0 h	0 (Control)	0P (Control)	44.73 ± 7.49	120.74 ± 18.01	628.03 ± 132.90 ab	25.63 ± 1.81 ab	18.94 ± 2.63	90.86 ± 23.51 ab	19.95 ± 8.18 f	106.76 ± 14.46 ab
	60 MPa	CUT	47.46 ± 5.06	91.19 ± 6.27	410.69 ± 43.41 b	21.84 ± 0.86 b	14.84 ± 0.57	58.22 ± 6.31 b	54.48 ± 11.41 ef	89.02 ± 13.76 b
		2P	46.13 ± 4.30	87.14 ± 9.01	553.99 ± 147.25 ab	24.68 ± 2.35 ab	15.75 ± 0.67	68.19 ± 27.12 b	248.41 ± 51.85 abcd	99.31 ± 10.81 b
		3P	35.58 ± 4.14	88.23 ± 8.50	657.26 ± 163.47 ab	29.03 ± 1.32 ab	19.59 ± 2.50	151.35 ± 53.47 ab	296.92 ± 68.43 ab	157.15 ± 11.84 ab
		4P	53.27 ± 8.47	104.80 ± 10.97	1156.12 ± 170.08 a	26.54 ± 0.76 ab	22.96 ± 2.64	208.33 ± 26.44 a	287.61 ± 62.17 abc	85.87 ± 9.89 b
		1P/5 min	51.11 ± 3.51	94.01 ± 13.45	1026.06 ± 146.64 ab	25.04 ± 1.94 ab	16.90 ± 2.18	144.05 ± 23.26 ab	274.54 ± 31.45 abcd	130.95 ± 20.27 ab
	100 MPa	CUT	46.49 ± 3.78	85.30 ± 12.89	719.55 ± 89.53 ab	25.63 ± 0.96 ab	18.33 ± 1.32	137.98 ± 15.60 ab	182.71 ± 14.82 bcde	139.59 ± 7.72 ab
		2P	67.16 ± 3.66	119.78 ± 13.33	781.40 ± 118.00 ab	25.57 ± 1.83 ab	18.62 ± 2.34	103.99 ± 17.84 ab	122.79 ± 8.62 def	137.58 ± 30.13 ab
		3P	78.59 ± 18.70	141.73 ± 27.03	914.63 ± 152.49 ab	32.83 ± 4.51 a	20.86 ± 3.47	163.61 ± 31.93 ab	368.67 ± 62.10 a	178.56 ± 17.29 a
		4P	41.21 ± 2.51	133.79 ± 20.54	650.48 ± 92.50 ab	27.22 ± 1.05 ab	16.15 ± 1.34	119.48 ± 20.99 ab	320.18 ± 103.78 ab	89.33 ± 13.42 b
		1P/5 min	51.20 ± 6.60	111.03 ± 14.98	637.86 ± 123.17 ab	28.32 ± 2.06 ab	24.24 ± 3.71	109.79 ± 66.48 ab	132.48 ± 64.62 cdef	95.17 ± 14.49 b
48 h	0 (Control)	0P (Control)	70.69 ± 9.27	149.06 ± 29.39 ab	923.22 ± 80.46	28.89 ± 1.43	26.36 ± 3.32	115.36 ± 31.69 b	183.99 ± 62.15 +abc	170.16 ± 33.25
	60 MPa	CUT	89.32 ± 14.61 *+	164.17 ± 40.53 ab	981.68 ± 220.40	28.40 ± 2.49	35.37 ± 7.09 +	200.04 ± 29.41 *+ab	425.94 ± 105.48 *+ab	194.94 ± 47.10 +
		2P	64.71 ± 7.66	97.61 ± 9.30 a	687.22 ± 136.93	29.81 ± 0.82	17.08 ± 2.09	98.04 ± 9.62 b	235.07 ± 43.29 *+abc	171.27 ± 21.08 *+
		3P	54.81 ± 6.34 +	102.44 ± 8.99 ab	883.63 ± 279.16	33.73 ± 4.53	21.91 ± 4.47	192.68 ± 62.49 ab	260.46 ± 64.07 *+abc	163.16 ± 39.54
		4P	111.69 ± 18.03 *+	158.84 ± 34.29 a	1079.89 ± 182.30	33.95 ± 2.6	31.33 ± 6.22	134.10 ± 17.11 +ab	242.07 ± 75.61 *abc	129.43 ± 9.25 +
		1P/5 min	56.77 ± 8.33	102.30 ± 13.49 ab	537.68 ± 129.21 +	26.16 ± 0.97	19.07 ± 2.42	102.45 ± 8.30 b	202.45 ± 32.13 *abc	93.17 ± 9.43
	100 MPa	CUT	55.57 ± 12.77	154.71 ± 23.84 +ab	983.54 ± 162.84	27.89 ± 1.82	24.66 ± 3.90	250.00 ± 49.03 *+a	435.43 ± 110.56 *+a	91.72 ± 23.16
		2P	70.06 ± 13.38	116.40 ± 13.59 ab	571.27 ± 51.77	27.01 ± 0.91	16.80 ± 2.18	131.93 ± 13.34 ab	229.25 ± 51.12 *abc	105.60 ± 8.48
		3P	66.79 ± 9.21	93.17 ± 13.38 ab	856.38 ± 126.86	31.00 ± 1.82	21.26 ± 2.44	152.21 ± 17.28 ab	260.44 ± 27.48 *abc	131.42 ± 27.75
		4P	49.91 ± 9.39	94.23 ± 14.39 ab	933.53 ± 154.25	30.65 ± 2.22	19.57 ± 2.02	122.15 ± 16.78 b	178.65 ± 49.59 *c	147.24 ± 18.47 +
		1P/5 min	45.95 ± 3.82	77.42 ± 8.86 b	708.68 ± 75.27	29.85 ± 2.72	15.67 ± 2.30	119.94 ± 8.14 b	190.64 ± 32.32 *bc	121.94 ± 12.17

^1^ Values are reported as 5-*O*-CQA equivalents for 3-*O*-CQA and as chlorogenic acid equivalents for 5-*O*-CQA. ^2^ Compounds were quantified at 280 nm (GA hex, 3- *O*-CQA, 3,5-diCQA, and isocoumarin) and at 320 nm (5-*O*-CQA, 4,5-diCQA, ferulic acid, and 3,4-diFQA). ^3^ Data represent the mean of three replicates ± standard error of the mean. ^4^ Different letters among treatments and storage times indicate values with a significant statistical difference by the Tukey test (*p* < 0.05). ^5^ Asterisk (*) Indicates statistical difference when comparing values of each stored sample against time 0 h control samples by the Student’s *t*-test (*p* < 0.05). ^6^ (+) Indicates statistical difference when comparing values of each stored sample against time 0 h HHP treated samples by the Student’s *t*-test (*p* < 0.05). ^7^ Abbreviations: GAH hex = gallic acid hexoside, 3-*O*-CQA = 3-*O*-caffeoylquinic acid, 5-*O*-CQA = 5-*O*-caffeoylquinic acid, 3,5-diCQA = 3,5-di caffeoylquinic acid, 4,5-diCQA = 4,5-di caffeoylquinic acid, and 3,4-diFQA = 3,4-di-feruloyquinic acid.

**Table 2:** Effect of static and multi-pulsed mild intensity pressure treatments on the concentration of bound phenolics in whole carrot samples.

Treatment			Phenolic Concentration (mg/kg DW) ^1,2,3,4,5,6,7^						
Storage Time	Pressure Applied (MPa)	Number Pulses	Caffeoyl Glucose	4-*O*-CoQA	5-*O*-CoQA	Caffeic Acid	*p*-Coumaric Acid	Rutin	Quercetin
0 h	0 (Control)	0P (Control)	14.98 ± 4.78	1.34 ± 0.25	15.68 ± 5.52 a	84.14 ± 13.19 ab	81.98 ± 9.12 c	8.42 ± 3.54 a	27.32 ± 7.36
	60 MPa	CUT	20.35 ± 0.75	2.12 ± 0.39	2.89 ± 0.07 b	72.46 ± 4.68 b	103.68 ± 6.46 bc	5.15 ± 0.42 ab	25.33 ± 3.32
		2P	19.46 ± 1.49	2.21 ± 0.48	4.02 ± 0.81 b	86.27 ± 11.06 ab	106.72 ± 9.69 bc	5.89 ± 1.26 b	12.93 ± 2.23
		3P	17.93 ± 1.59	3.47 ± 0.80	5.19 ± 1.40 b	112.24 ± 12.45 ab	175.58 ± 25.25 ab	6.24 ± 2.35 ab	26.99 ± 4.05
		4P	25.30 ± 2.82	2.55 ± 0.27	7.55 ± 0.65 ab	114.03 ± 9.42 ab	184.22 ± 22.56 a	9.38 ± 1.80 ab	27.52 ± 0.61
		1P/5 min	20.91 ± 2.45	3.06 ± 0.63	4.13 ± 0.84 b	104.03 ± 11.38 ab	149.66 ± 14.91 abc	5.06 ± 0.94 ab	17.39 ± 2.32
	100 MPa	CUT	17.27 ± 2.73	1.34 ± 0.60	2.88 ± 0.53 b	75.97 ± 5.61 ab	113.48 ± 14.37 bc	3.07 ± 0.65 ab	14.04 ± 2.27
		2P	24.36 ± 1.68	2.14 ± 0.33	4.13 ± 0.94 b	85.14 ± 3.62 ab	108.09 ± 11.44 bc	4.67 ± 0.28 ab	16.18 ± 2.64
		3P	18.25 ± 3.78	1.87 ± 0.38	4.08 ± 0.87 b	87.55 ± 8.40 ab	112.88 ± 17.14 bc	6.89 ± 1.29 ab	25.09 ± 3.58
		4P	22.49 ± 5.01	2.03 ± 0.32	6.53 ± 2.50 ab	93.58 ± 2.73 a	141.01 ± 17.72 abc	4.64 ± 1.89 ab	15.82 ± 2.63
		1P/5 min	14.91 ± 1.20	2.08 ± 0.30	4.50 ± 0.62 b	89.73 ± 2.28 ab	113.51 ± 3.47 bc	8.53 ± 2.65 ab	17.87 ± 1.44
48 h	0 (Control)	0P (Control)	17.20 ± 3.39	2.03 ± 0.24 **+**	10.89 ± 3.31 a	134.55 ± 14.51 **+**ab	135.85 ± 16.81 **+**	9.34 ± 2.11	20.63 ± 4.28
	60 MPa	CUT	23.24 ± 5.65	2.08 ± 0.35	4.65 ± 1.11 ab	95.41 ± 8.05 **+**bc	143.49 ± 32.87	5.87 ± 1.50	29.93 ± 3.98 **+**
		2P	16.72 ± 1.60	2.37 ± 0.53	2.61 ± 0.48 b	118.02 ± 10.21 abc	128.70 ± 24.35	4.38 ± 0.58 **+**	20.75 ± 2.34
		3P	15.90 ± 2.18	3.36 ± 0.97 *	4.12 ± 1.12 ab	127.15 ± 13.91 abc	162.65 ± 17.18 *	8.01 ± 2.39	25.27 ± 4.87
		4P	19.61 ± 1.78	2.14 ± 0.44 **+**	5.04 ± 0.99 ab	117.70 ± 8.67 abc	107.08 ± 16.55 **+**	6.14 ± 0.90 **+**	21.05 ± 2.33 **+**
		1P/5 min	19.76 ± 2.24	3.05 ± 0.52 *	2.89 ± 0.46 b	126.13 ± 16.88 abc	163.30 ± 21.17 *	5.61 ± 1.76	29.18 ± 1.99
	100 MPa	CUT	16.66 ± 2.10	2.26 ± 0.31 *+	5.61 ± 1.51 ab	130.15 ± 5.07 *+abc	146.08 ± 17.44 *	6.76 ± 1.92 **+**	26.53 ± 110.56
		2P	18.42 ± 2.39 **+**	1.73 ± 0.48 **+**	5.96 ± 2.36 ab	76.91 ± 11.61 c	119.67 ± 12.09 *	2.48 ± 0.25	19.24 ± 51.12
		3P	25.34 ± 3.25	1.72 ± 0.17	5.00 ± 0.51 ab	112.21 ± 13.01 abc	145.03 ± 15.22 *	4.26 ± 0.75	19.71 ± 27.48
		4P	27.23 ± 6.05	2.48 ± 0.53	6.09 ± 1.19 ab	132.44 ± 8.58 *+ab	117.96 ± 14.78 *	5.60 ± 2.77	32.70 ± 49.59
		1P/5 min	27.71 ± 4.05 *+	2.19 ± 0.47	3.80 ± 0.45 ab	165.46 ± 4.85 *+a	172.22 ± 9.14 **+**	7.39 ± 1.44	25.76 ± 3.90

^1^ Values are reported as 5-*O*-CQA equivalents for caffeoyl glucose and as *p*-coumaric acid equivalents for 4-*O*-CoQA and 5-*O*-CoQA. ^2^ Compounds were quantified at 280 nm (caffeoyl glucose, 5-*O*-CoQA and caffeic acid) and at 320 nm (4-*O*-CoQA, *p*-coumaric acid, rutin and quercetin). ^3^ Data represent the mean of three replicates ± standard error of the mean. ^4^ Different letters among treatments and storage times indicate values with a significant statistical difference by the Tukey test (*p* < 0.05). ^5^ Asterisk (*) Indicates statistical difference when comparing values of each stored sample against time 0 h control samples by the Student’s *t*-test (*p* < 0.05). ^6^ (+) Indicates statistical difference when comparing values of each stored sample against time 0 h HHP treated samples by the Student’s *t*-test (*p* < 0.05). ^7^ Abbreviations: 4-*O*-CoQA = 4-*O*-coumaroylquinic acid, 5-*O*-CoQA= 5-*O*-coumaroylquinic acid, 5-*O*-CQA = 5-*O*-caffeoylquinic acid.

**Table 3:** Effect of static and multi-pulsed mild intensity pressure treatments on the concentration of carotenoids in whole carrot samples.

Treatment			Carotenoids (mg/kg DW) ^1,2,3,4,5,6^					
Storage Time	Pressure Applied (MPa)	Number of	All-*trans*-Lutein	All-*trans*-Zeaxanthin	All-*trans*-β-	All-*trans-*α-Carotene	All-*trans-*β-Carotene	RAE
		Pulses			Cryptoxanthin			
0 h	0 (control)	0P (control)	243.73 ± 14.46 bc	210.36 ± 14.54 b	271.00 ± 13.39 b	2132.60 ± 314.06 a	3449.22 ± 590.35 ab	777.95 ± 125.48
	60 MPa	CUT	246.38 ± 2.72 ab	392.70 ± 5.99 a	476.40 ± 13.28 a	1787.24 ± 249.39 ab	2940.72 ± 438.25 ab	731.61 ± 87.69
		2P	261.16 ± 11.06 abc	370.76 ± 11.31 a	455.70 ± 11.02 a	2017.37 ± 209.58 a	3211.89 ± 185.84 ab	738.01 ± 43.86
		3P	289.14 ± 29.15 a	360.99 ± 22.35 a	401.09 ± 32.52 ab	1837.84 ± 197.90 ab	3136.04 ± 432.58 ab	706.49 ± 85.47
		4P	274.06 ± 9.04 ab	327.55 ± 25.25 ab	401.41 ± 22.98 ab	2236.62 ± 176.96 a	3817.81 ± 454.93 a	852.98 ± 87.88
		1P/5 min	263.64 ± 8.85 abc	364.32 ± 8.54 a	447.34 ± 15.47 a	1789.41 ± 325.64 ab	3213.17 ± 583.92 ab	617.90 ± 85.36
	100 MPa	CUT	264.30 ± 10.15 abc	369.65 ± 3.47 a	451.69 ± 9.14 a	1597.92 ± 206.31 ab	2969.69 ± 426.48 ab	661.54 ± 86.53
		2P	237.74 ± 8.99 c	367.84 ± 10.83 a	448.67 ± 13.81 ab	1307.35 ± 257.71 b	2227.93 ± 448.67 b	603.99 ± 41.78
		3P	267.99 ± 10.36 abc	360.26 ± 24.91 a	460.94 ± 24.77 a	2080.14 ± 216.03 a	3793.47 ± 440.44 a	839.73 ± 88.89
		4P	264.98 ± 6.83 abc	363.50 ± 13.85 a	431.49 ± 21.41 ab	2179.78 ± 219.49 a	3327.18 ± 423.18 ab	768.41 ± 86.75
		1P/5 min	267.54 ± 7.88 abc	374.09 ± 14.93 a	443.67 ± 21.57 a	2031.51 ± 195.60 a	3523.55 ± 348.88 a	790.54 ± 72.87
48 h	0 (control)	0P (control)	253.17 ± 15.93 b	269.17 ± 26.00 b	336.12 ± 17.77 b	2005.70 ± 147.78	3312.19 ± 216.25	747.15 ± 45.08 ab
	60 MPa	CUT	264.85 ± 2.32 ab	382.74 ± 5.96 *a	441.05 ± 13.19 *a	1993.01 ± 153.23	3326.14 ± 314.02	755.28 ± 63.50 ab
		2P	267.07 ± 4.35 ab	368.81 ± 22.58 *ab	446.36 ± 27.55 *ab	1912.55 ± 123.58	3413.85 ± 319.08	762.46 ± 61.25 ab
		3P	282.93 ± 12.67 a	373.49 ± 10.18 *ab	462.46 ± 2.72 *ab	2067.26 ± 154.59	3652.17 ± 280.34	814.99 ± 56.85 ab
		4P	258.96 ± 8.16 ab	333.30 ± 13.88 *ab	394.64 ± 20.21 *ab	1695.46 ± 299.54	2924.97 ± 640.29	660.18 ± 130.74 b
		1P/5 min	261.44 ± 6.39 ab	377.43 ± 11.03 *ab	448.45 ± 33.12 *a	1987.44 ± 131.55	3366.14 ± 207.60	761.31 ± 44.91 ab
	100 MPa	CUT	268.12 ± 13.35 ab	356.08 ± 21.14 *ab	436.85 ± 33.45 *ab	1942.96 ± 224.74	3394.57 ± 389.80	744.38 ± 84.04 ab
		2P	263.52 ± 8.78 ab	384.71 ± 8.28 *a	446.20 ± 11.04 *a	1886.07 ± 283.41	3042.75 ± 566.02	698.94 ± 116.10 ab
		3P	270.17 ± 8.21 ab	356.50 ± 21.10 *ab	438.94 ± 38.86 ab	2148.09 ± 154.03	3983.80 ± 348.23	875.35 ± 69.81 a
		4P	262.30 ± 9.42 ab	382.49 ± 11.71 *ab	480.06 ± 26.99 *a	1853.20 ± 324.30	3463.87 ± 629.12	767.82 ± 129.06 ab
		1P/5 min	251.61 ± 4.67 b	384.39 ± 9.49 *a	464.10 ± 11.77 *a	1677.95 ± 223.94	2831.16 ± 420.47	647.13 ± 85.87 ab

^1^ Values are reported as all-*trans*-lutein equivalents for all-*trans*-zeaxanthin, all-*trans*-β-cryptoxanthin and as all-*trans*-β-carotene acid equivalents for all-*trans*-α-carotene. ^2^ All compounds were quantified at 450 nm. ^3^ Values represent the mean of three replicates ± standard error of the mean. ^4^ Different letters among treatments and storage times indicate values with significant statistical difference by the Tukey test (*p* < 0.05). ^5^ Asterisk (*) Indicates statistical difference when comparing values of each stored sample against time 0 h control samples by the Student’s *t*-test (*p* < 0.05). ^6^ Abbreviations: RAE, retinol activity equivalents.
